# Study on the Difference of BIS/BAS Scale between Sasang Types

**DOI:** 10.1155/2015/805819

**Published:** 2015-11-19

**Authors:** Soo Jin Lee, Sung Hye Kim, Nangyeon Lim, Mi Young Ahn, Han Chae

**Affiliations:** ^1^Department of Psychotherapy, School of Nursing and Public Health, Kyungil University, Daegu 38428, Republic of Korea; ^2^Division of Longevity and Biofunctional Medicine, School of Korean Medicine, Pusan National University, Busan 50610, Republic of Korea; ^3^Department of Social Welfare, School of Humanities and Social Science, Youngdong University, Chungbook 29131, Republic of Korea

## Abstract

*Introduction*. The purpose of this study was to examine the differences in temperament between So-Yang (SY) and So-Eum (SE) Sasang types using Behavioral Inhibition System/Behavioral Activation System (BIS/BAS) scale to elucidate the biopsychological mechanism underlying the Sasang typology, a traditional Korean personalized medicine. *Methods*. 248 university students were categorized into three Sasang types, and series of *t*-tests were conducted, separately for male and female participants, to examine the difference of Behavioral Inhibition System (BIS), Behavioral Activation System (BAS), BAS-Drive (BAS-D), BAS-Fun Seeking (BAS-FS), and BAS-Reward Responsiveness (BAS-RR) scores between SY and SE Sasang types. *Results*. There were significant differences between Sasang types in the BIS/BAS subscales with consideration of gender. In male participants, BAS-total score of SY type (39.75 ± 4.56) was significantly (*t* = 2.462, *p* = 0.016) higher than that of SE type (36.68 ± 4.97). On the other hand, in female participants, BIS score of SY type (20.10 ± 4.01) was significantly (*t* = −2.097, *p* = 0.039) lower than that of SE type (21.83 ± 3.91). *Discussion*. The current study suggested relationship between Sasang typology and Behavior Inhibition and Activation Systems and showed significant differences in BIS/BAS scale between SY and SE Sasang types. Further studies on biological base of Sasang typology are needed.

## 1. Introduction

Sasang typology is a traditional Korean medical typology dividing people into four Sasang types based on their unique organ system [1], which determines type-specific temperaments [2, 3], pathophysiological characteristics [4], disease susceptibility [5, 6], and response to specific treatments [7]. Research has been reported that the psychobiological characteristics of Sasang typology might have biological basis [8–10]; however it was not satisfactory to indicate underlying mechanisms (Table 1).

The So-Yang Sasang type is an extroverted, active, inquisitive, outgoing, quick-tempered, excitable, dynamic, easy-going, and impulsive person with strong interest in the outside world; on the contrary, the So-Eum Sasang type is introverted, passive, negative, organized, reserved, static, meticulous, patient, cautious, and nervous person focusing on their inner world [8].

Previous studies on the temperament of each Sasang type presented that the characteristics of So-Yang (SY) and So-Eum (SE) Sasang types are opposing each other [2, 3, 8, 11]. It has been reported that the So-Yang and So-Eum types have contrasting characteristics with Eysenck's Neuroticism and Extraversion and Cloninger's Novelty-Seeking (NS) and Harm-Avoidance (HA) [2]. The SY type was high on Extraversion and NS whereas it was low on Neuroticism and HA. On the contrary, the SE type was high on Neuroticism and HA whereas it was low on Extraversion and NS [2, 3, 8].

Interestingly, Cloninger's NS and HA are known to have influence from Gray's Behavioral Activation and Inhibition System, which might have important meanings in Sasang typology, equivalently [10, 12]. Gray's Behavioral Activation and Inhibition System are two pivotal motivational systems, which are considered to be responsible for affective states, behavior, personality, and predispositions for various forms of psychopathology, and there have been considerable number of studies for the measurement and understanding of its neuroanatomical and psychometric properties [13–15].

Considering the meanings of inhibition and activation from biological perspective, these two contrasting concepts can be translated into Yin and Yang of traditional medicine in dualistic perspective. Yin-Yang is a representative term for two opposite characteristics of nature as introvert-extrovert, passive-active, negative-positive, cold-hot, moon-sun, night-day, dark-bright, slow-fast, and so on [8].

As for a biological system, Yang is predisposed to respond actively and externally towards environmental stimuli, whereas Yin is predisposed to be withdrawn and passive. Comparing the concept of Yin-Yang in traditional medicine to Western biological concepts of Behavioral Inhibition and Activation is an interesting research topic, in that it attempts an integrated approach toward research on biopsychology across the East and the West.

Therefore, in the current study, it was aimed to test whether the Behavioral Activation and Inhibition systems reflect the characteristics of SY and SE Sasang types, which represents the biological characteristics of Yang and Yin [8], respectively. Carver and White's BIS/BAS scale was used for the measure of Behavioral Activation and Inhibition system [16, 17]. It measures Behavioral Inhibition System (BIS) and Behavioral Activation System (BAS). The BIS measures the activation from aversive stimuli such as anxiety, fear, and worry. The BAS is a sum of three subscales of BAS-Drive (BAS-D), BAS-Fun Seeking (BAS-FS), and BAS-Reward Responsiveness (BAS-RR). The BAS-D measures the degree to which individuals pursue appetitive goals. BAS-FS reflects tendency to seek new potentially rewarding experiences and to act on incentives of the moment. BAS-RR focuses on positive responses to an occurrence of reward.

The results in this study would be useful for analyzing biopsychological characteristics of Sasang typology and would provide new diagnostic tools for those who have interest in differentiating Sasang types in their clinics. Furthermore, we can find similarities and differences between traditional temperament theory of Yin and Yang and Western biopsychology of BAS and BIS, and it would be useful for providing foundations for integrative biopsychology across the East and the West [8, 12].

## 2. Methods

### 2.1. Participants and Measurement

A total of 270 individuals from School of Korean Medicine were asked to complete QSCCII for Sasang type classification and BIS/BAS scale for BAS-D, BAS-FS, BAS-RR, BAS, and BIS measurement. The following procedures were approved by the Institutional Review Board of Pusan National University. All participants were provided with a written consent form for this study.

The BIS/BAS scale was developed by Carver and White [16] and was tested for the psychometric structure [18, 19]. We used Korean version translated by K.-H. Kim and W.-S. Kim [17], which was reaffirmed by comparing the original and translated versions side-by-side. The BIS/BAS scale consists of 24 items including four filler items, and each item is scored using a 4-point Likert scale from “strongly disagree” [1] to “strongly agree” [4]. The BAS scale has 13 items including BAS-D (4 items), BAS-FS (4 items), and BAS-RR (5 items), whereas the BIS has 7 items.

The QSCCII, a Sasang typology-based diagnostic inventory, is composed of 121 items concerning type-specific body shapes, psychological characteristics, life style, and pathophysiological symptoms. It was developed in 1993 and was revised in 1996. It has been used as an objective measurement in Sasang typology studies. The correctly predicted percentage of QSCCII was reported as 70.08% [20]; and the internal consistency calculated with Cronbach's alpha for Tae-Yang (TY), So-Yang (SY), Tae-Eum (TE), and So-Eum (SE) Sasang types was 0.57, 0.58, 0.59, and 0.63, respectively [21].

### 2.2. Statistical Analysis

Descriptive statistics on gender, education level, and age of each Sasang type were analyzed. *χ* ^2^ test for gender and education level and Analysis of Variance (ANOVA) for age were used to find differences between Sasang types.

The internal consistency of BIS/BAS subscales was analyzed with Cronbach's alpha. Item number and mean and standard deviation for each subscale were calculated.

*t*-test was conducted to compare BAS-D, BAS-FS, BAS-RR, BAS, and BIS levels between SY and SE types. Statistical results were presented as frequency (%) or mean ± standard deviation, and statistical significance level was set at *p* < 0.05, *p* < 0.01, and *p* < 0.001. PASW Statistics 18.0 (IBM, Armonk, NY) was used for all statistical analysis.

## 3. Results

### 3.1. Demographic Characteristics of the Participants

Data from 248 participants were used in the final analysis. The numbers of So-Yang (SY), Tae-Eum (TE), and So-Eum (SE) types classified with QSCC II were 58, 64, and 126, respectively (Table 2). As a result of the analyses to find differences of Sasang types between groups, there was a significant difference of Sasang type between genders (*χ* ^2^ = 11.960, *p* = 0.03). However, no significant difference was found in age or education level.

### 3.2. Reliability of the BIS/BAS Scale

Internal consistencies of BIS and BAS items were calculated with Cronbach's alpha (Table 3). Data from 247 participants were used. The mean and standard deviation of BAS (13 items) were 38.17 ± 27.94, whereas the mean and standard deviation of BIS (7 items) were 20.09 ± 14.20. Cronbach's alpha levels were acceptable: 0.824 and 0.834 for BAS and BIS, respectively.

### 3.3. BIS/BAS Scale Profile of So-Yang and So-Eum Sasang Types in Male and Female Participants

In male participants, the results of *t*-test to evaluate the differences between SY and SE types are shown in Table 4. The differences of BAS-D and BAS between SY and SE types were significant. The BAS-D of SY type (11.70 ± 1.94) was significantly (*t* = 2.216, *p* = 0.029) higher than that of SE type (10.50 ± 2.15). The BAS of SY type (39.75 ± 4.56) was significantly (*t* = 2.462, *p* = 0.016) higher than that of SE type (36.68 ± 4.97).

In female participants, the results of *t*-test to find the differences between SY and SE types were shown in Table 5. The differences of BAS-FS and BIS between SY and SE types were significant. The BAS-FS of SY type (12.15 ± 1.96) was significantly (*t* = 2.201, *p* = 0.030) higher than that of SE type (11.11 ± 2.44). On the other hand, the BIS of SY type (20.10 ± 4.01) was significantly (*t* = −2.097, *p* = 0.039) lower than that of SE type (21.83 ± 3.91).

## 4. Discussion

The current study examined Carver and White's BIS/BAS scale profile of SY and SE Sasang types (Table 1) and showed that the BAS of male participants and the BIS of female participants were significantly different between SY and SE Sasang types (Tables 4 and 5).

The results in this study, in combination with previous studies with Cloninger's NS and HA, are partly confirmed as hypothesized, in that the mechanism of Sasang typology might be related to the neurobiological base of Gray's biological personality theory [10]. Gray's notion of Behavioral Activation and Inhibition Systems suggests two main brain systems regulating approach and withdrawal behaviors to environmental stimuli [8, 22].

The Yin and Yang (Eum and Yang) are two opposing and complementary sides of the nature and have been a theoretical basis of traditional medicine for thousands of years in East [8]. And this concept was shown to have phenotypic similarities with temperament (cold-warm) and humidity (wet-dry) in humoral theory of Hippocrates and Galen which is an archetype of Western personality theories [23].

It was reported in functional neuroscience literature that the differential activation patterns in hippocampus, parahippocampal cortex, and amygdala caused by fear-related stimuli are positively correlated with Carver and White's BIS [24], which was shown to play a role in modulating anxiety related behaviors and negative emotion [22]. And the mesolimbic and mesocortical dopamine projections including ventral striatum and orbital regions of the prefrontal cortex are shown to be related to Carver and White's BAS [25, 26], which is known to be crucial for approach behavior and reward-related motivation [22].

Considering existing research on the biological base of Sasang typology along with current study [2, 3, 8, 9], SY and SE Sasang type might have differences in Behavioral Activation and Inhibition system, which have neuroanatomical structural basis. Those with SE Sasang type might have anxiety-related and negative emotion-related serotonergic circuits in hippocampus, parahippocampal cortex, and amygdala that have innate tendency to show negative or avoidant reaction to the unknown or harmful stimulations. SY Sasang type might have predisposed development with approach and reward-related mesolimbic and mesocortical dopamine projections including ventral striatum and orbital regions of the prefrontal cortex which could result in active and explorative reaction to the outside environment [8, 10, 12, 22]. Further studies on the relationships of SY and SE Sasang types with neuronal structure and neurotransmitter would be needed.

As for the reason that there were gender differences in BIS and BAS scale, unlike the case with Cloninger's NS and HA, there would be some speculations. First, the psychometric structure of Carver and White's BIS/BAS scale might include gender differences, which was shown as pivotal for Sasang typology [8]. Carver and White previously reported that BIS and BAS-RR of female participants (21.09 and 17.90, resp.) were significantly higher than those of male participants (18.84 and 17.27, resp.) [16]. The gender-specific neuroanatomical basis of BIS/BAS scale [22] and the relationship between BIS/BAS scale and socioemotional functioning in children [27] were also reported.

Second, there is a possibility that some items of BIS/BAS scale are related to gender roles differentially applied in the East and the West [8]. Though the filler items are not included in the calculation, the following items might trigger gender roles in East Asian society: “a person's family is the most important thing in life,” “how I dress is important to me,” and “it is hard for me to find the time to do things such as getting a haircut.”

Third, the BIS/BAS scale may measure different dimensions from those of Cloninger's HA and NS, which was reported to measure Gray's Behavioral Activation and Inhibition System [28, 29]. As an example, when correlation analysis was conducted between BIS/BAS and Tridimensional Personality Questionnaire (TPQ), Cloninger's previous biopsychological model of TCI, HA (*r* = 0.59, *p* < 0.001) was significantly correlated with BIS but NS (*r* = −0.11,* n*.*s*.) was not [16].

These results emphasize a need for additional correlation studies on the personality construct of BIS/BAS and TCI along with Sasang Personality Questionnaire (SPQ), which measures the temperamental dimension of Sasang typology. The SPQ is a Yin-Yang based objective dimensional measurement with proven clinical validity and reliable psychometric properties [8]. The SPQ-total score showed distinctive differences between SY and SE types with respect to age and gender [8].

Considering that the BIS/BAS scale can measure the differences between SY and SE Sasang types, the Sasang typology can be used in diverse clinical fields since its Korean BIS/BAS version [17] has been used for the studies on heart rate variability [30], eating behavior [31], internet game addiction [32], depression [33], problematic alcohol use [34], motivation and interest [35], response to affective stimuli [36], and subjective well-being [37].

The results of the current study supported our prediction that significant differences of BIS and BAS exist between SY and SE Sasang types when gender is taken into account. The understanding for biological basis of Sasang typology would become more profound and integral with further research incorporating more participants and cultural contexts [38].

## Figures and Tables

**Table 1:** Characteristics of So-Yang and So-Eum Sasang types.

Sasang type (prevalence)	So-Yang (*소양*, 少陽) (30%)	So-Eum (*소*음, *少陰*) (30%)
Origin of the nature	Anger (怒) by righteousness (義)	Enjoyment (樂) by wisdom (智)
	They become angry when they are blocked. The anger can be regulated by fairness	Worries can be relieved with wisdom. They enjoy what they have now

Temperament or personality characteristics	Active, externally oriented, and talented for business.	Still, internally oriented, and self-directed
	Unstable, easily getting bored, sacrificing, righteous, easily acceptable, quick tempered, active, and easy-going	Neat, mild, negative, intelligent, organized, patient, jealous, perseverant, passive, static, and meticulous
	High Extraversion (NEO-PI). High Novelty-Seeking and low Harm-Avoidance (TCI). High SPQ	Low Extraversion. Low Novelty-Seeking and high Harm-Avoidance. Low SPQ. Low Positive Affect (PANAS). High in Trait Anxiety (STAI)

Pathophysiological characteristics	Strong intake and digestion and weak waste discharge	Strong waste discharge and weak intake and digestion

Concerns for the good health	Easy with defecation	Good digestion
	Avoid overactivation and overloads of bodily functions	Maintain healthy digestive function, peristalsis, and body heat

Frequent symptoms or disease	Constipation, gastroesophageal (laryngopharyngeal) reflux disease, affective disorder, insomnia, and heat on chest	Indigestion or dyspepsia, upper respiratory infection, and neurotic symptoms

Type-specific medical herbs	Rehmanniae Radix, Corni Fructus, Hoeoen, Alismatis Rhizoma, Osterici Radix, and Angelicae Pubescentis Radix	Ginseng Radix, Atractylodis Rhizoma Alba, Glycyrrhizae Radix, Cinnamomi Cortex, Citri Pericarpium, Zingiberis Rhizoma Crudus

Type-specific acupuncture points	Diagnosis with HT3. Treatment with HT7(+)/SP3(−)	Diagnosis with HT7. Treatment with SP3(+)/LI4(−)

TCI: Temperament and Character Inventory; NEO-PI: NEO Personality Inventory; SPQ: Sasang Personality Questionnaire; PANAS: Positive and Negative Affect Schedule; STAI: State and Trait Anxiety Inventory.

**Table 2:** Demographic features of the subjects in this study.

	So-Yang	Tae-Eum	So-Eum	
Sex^*∗*^				Chi-square = 11.960, *p* = 0.03
(male/female)	20/38	42/22	67/59	

Age	28.53 ± 3.21	28.62 ± 3.64	29.00 ± 5.52	*F* = 0.259, *p* = 0.772

Education				Chi-square = 1.874, *p* = 0.392
Bachelor	41	52	95	
Master	17	12	31	

^*∗*^ *p* < 0.05.

**Table 3:** Internal Consistency of the BIS/BAS scale.

	# of items	Mean	St. dev.	Cronbach's alpha
BAS	13	38.17	27.94	0.824
BAS-D	4	10.90	4.41	0.701
BAS-FS	4	11.25	5.26	0.705
BAS-RR	5	16.02	5.03	0.723
BIS	7	20.09	14.20	0.834

BAS: Behavior Activation System; BAS-D: BAS-Drive; BAS-FS: BAS-Fun Seeking; BAS-RR: BAS-Reward Responsiveness; BIS: Behavior Inhibition System.

**Table 4:** Mean and SD of BIS/BAS subscales in male So-Yang (*n* = 20) and So-Eum (*n* = 67) Sasang type groups.

	So-Yang	So-Eum	
BAS^*∗*^	39.75 ± 4.56	36.68 ± 4.97	*t* = 2.462, *p* = 0.016
BAS-D^*∗*^	11.70 ± 1.94	10.50 ± 2.15	*t* = 2.216, *p* = 0.029
BAS-FS	11.70 ± 2.10	10.76 ± 2.22	*t* = 1.672, *p* = 0.098
BAS-RR	16.35 ± 1.98	15.41 ± 2.03	*t* = 1.81, *p* = 0.074
BIS	19.05 ± 3.11	19.86 ± 3.23	*t* = −0.996, *p* = 0.322

^*∗*^ *p* < 0.05.

BAS: Behavior Activation System; BAS-D: BAS-Drive; BAS-FS: BAS-Fun Seeking; BAS-RR: BAS-Reward Responsiveness; BIS: Behavior Inhibition System.

**Table 5:** Mean and SD of BIS/BAS subscales in female So-Yang (*n* = 38) and So-Eum (*n* = 59) Sasang type groups.

	So-Yang	So-Eum	
BAS	39.57 ± 5.05	38.57 ± 5.11	*t* = 0.945, *p* = 0.347
BAS-D	11.00 ± 2.18	10.94 ± 1.99	*t* = 0.118, *p* = 0.906
BAS-FS^*∗*^	12.15 ± 1.96	11.11 ± 2.44	*t* = 2.201, *p* = 0.030
BAS-RR	16.42 ± 2.16	16.50 ± 2.20	*t* = −0.191, *p* = 0.848
BIS^*∗*^	20.10 ± 4.01	21.83 ± 3.91	*t* = −2.097, *p* = 0.039

^*∗*^ *p* < 0.05.

BAS: Behavior Activation System; BAS-D: BAS-Drive; BAS-FS: BAS-Fun Seeking; BAS-RR: BAS-Reward Responsiveness; BIS: Behavior Inhibition System.

## References

[1] Lee J. (1894). *Longevity and Life Preservation in Eastern Medicine*.

[2] Chae H., Park S. H., Lee S. J., Kim M.-G., Wedding D., Kwon Y.-K. (2009). Psychological profile of sasang typology: a systematic review. *Evidence-Based Complementary and Alternative Medicine*.

[3] Jung S.-A. (2015). Psychological typology of Sasang medicine. *Integrative Medicine Research*.

[4] Chae H., Kim S. H., Han S. Y. (2014). Study on the psychobiological characteristics of Sasang Typology based on the type-specific pathophysiological digestive symptom. *Korean Journal of Oriental Physiology & Pathology*.

[5] Lee J., Lee J., Lee E., Yoo J., Kim Y., Koh B. (2011). The sasang constitutional types can act as a risk factor for hypertension. *Clinical and Experimental Hypertension*.

[6] Lee T. G., Koh B., Lee S. (2009). Sasang constitution as a risk factor for diabetes mellitus: a cross-sectional study. *Evidence-Based Complementary and Alternative Medicine*.

[7] Lee M. S., Shin B.-C., Choi S.-M., Kim J. Y. (2009). Randomized clinical trials of constitutional acupuncture: a systematic review. *Evidence-Based Complementary and Alternative Medicine*.

[8] Lee S. J., Park S. H., Cloninger C. R., Kim Y. H., Hwang M., Chae H. (2014). Biopsychological traits of Sasang typology based on Sasang personality questionnaire and body mass index. *BMC Complementary and Alternative Medicine*.

[9] Chae H., Kown Y. (2015). Best-fit index for describing physical perspectives in Sasang typology. *Integrative Medicine Research*.

[10] Sung W. Y., Kim W. K., Song J. M., Kim L. H. (2012). Study on personality traits of Sasang constitution with TCI and EPQ. *Journal of Oriental Neuropsychiatry*.

[11] Lee S., Park S., Chae H. (2013). Study on the temperament construct of Sasang typology with biopsychological measures. *Korean Journal of Oriental Physiology & Pathology*.

[12] Lee S., Cloninger C. R., Cloninger K. M., Chae H. (2014). The temperament and character inventory for integrative medicine. *Journal of Oriental Neuropsychiatry*.

[13] Torrubia R., Ávila C., Moltó J., Caseras X. (2001). The Sensitivity to Punishment and Sensitivity to Reward Questionnaire (SPSRQ) as a measure of Gray's anxiety and impulsivity dimensions. *Personality and Individual Differences*.

[14] Heym N., Ferguson E., Lawrence C. (2008). An evaluation of the relationship between Gray's revised RST and Eysenck's PEN: distinguishing BIS and FFFS in Carver and White's BIS/BAS scales. *Personality and Individual Differences*.

[15] Keiser H. N., Ross S. R. (2011). Carver and Whites' BIS/FFFS/BAS scales and domains and facets of the Five Factor Model of personality. *Personality and Individual Differences*.

[16] Carver C. S., White T. L. (1994). Behavioral inhibition, behavioral activation, and affective responses to impending reward and punishment: the BIS/BAS Scales. *Journal of Personality and Social Psychology*.

[17] Kim K.-H., Kim W.-S. (2001). Korean-BAS/BIS scale. *The Korean Journal of Health Psychology*.

[18] Smits D. J. M., Boeck P. D. (2006). From BIS/BAS to the big five. *European Journal of Personality*.

[19] Segarra P., Poy R., López R., Moltó J. (2014). Characterizing Carver and White's BIS/BAS subscales using the Five Factor Model of personality. *Personality and Individual Differences*.

[20] Lee J. C., Koh B. H., Song I. B. (1996). The validation study of the questionnaire for sasang constitution classification (the 2nd edition revised in 1995)—in the field of profile analysis. *Journal of Sasang Constitutional Medicine*.

[21] Kim S. H., Koh B. H., Song I. B. (1996). A study on the standardization of QSCC II (Questionnaire for Sasang Constitution Classification II). *Journal of Sasang Constitutional Medicine*.

[22] Li Y., Qiao L., Sun J. (2014). Gender-specific neuroanatomical basis of behavioral inhibition/approach systems (BIS/BAS) in a large sample of young adults: a voxel-based morphometric investigation. *Behavioural Brain Research*.

[23] Park S. H., Kim M. G., Lee S. J., Kim J. Y., Chae H. (2011). Temperament and character profiles of sasang typology in an adult clinical sample. *Evidence-Based Complementary and Alternative Medicine*.

[24] Mathews A., Yiend J., Lawrence A. D. (2004). Individual differences in the modulation of fear-related brain activation by attentional control. *Journal of Cognitive Neuroscience*.

[25] Simon J. J., Walther S., Fiebach C. J. (2010). Neural reward processing is modulated by approach- and avoidance-related personality traits. *NeuroImage*.

[26] Bray S., Shimojo S., O'Doherty J. P. (2010). Human medial orbitofrontal cortex is recruited during experience of imagined and real rewards. *Journal of Neurophysiology*.

[27] Kingsbury A., Coplan R. J., Weeks M., Rose-Krasnor L. (2013). Covering all the BAS's: a closer look at the links between BIS, BAS, and socio-emotional functioning in childhood. *Personality and Individual Differences*.

[28] Cloninger C. R. (1987). A systematic method for clinical description and classification of personality variants: a proposal. *Archives of General Psychiatry*.

[29] Cloninger C. R., Svrakic D. M., Przybeck T. R. (1993). A psychobiological model of temperament and character. *Archives of General Psychiatry*.

[30] Kim W.-S., Jho M.-J., Kim K.-H., Yoon Y.-R. (2003). Effects of behavioral activation/inhibition systems and positive/negative affective sounds on heart rate variability. *Korean Journal of the Science of Emotion and Sensibility*.

[31] Park H. J., Kim K. H. (2007). The effects of restrained eating, behavioral activation system (BAS), and preloading on eating behavior. *The Korean Journal of Health Psychology*.

[32] Chang M. S., Kim H. M., Kim S. Y. (2013). The effects of behavioral activation system/behavioral inhibition system (BAS/BIS) on decision-making in internet game addict. *Korean Journal of Health Psychology*.

[33] Koh M. K., Kim H. S. (2013). Emotional inertia and depression: influence of behavioral activation and ways of stress coping. *The Korean Journal of Clinical Psychology*.

[34] Suh K. H., Kim S. M., Jeong G. C. (2006). Behavioral activation and inhibition system, gender, family alcohol use, motivation for alcohol use, and problematic alcohol use among college students. *Korean Journal of Health Psychology*.

[35] Suh K.-H. (2007). Behavioral activation system, physical activity and interest in physical activity among college students. *Journal of Korea Sport Research*.

[36] Yoon B.-S. (2010). BIS and BAS related difference on cognitive and psychophysiological responses to the affective stimuli. *Korean Journal of Psychology: General*.

[37] Suh K.-H., Kim J.-H., You J.-M. (2009). The relationship between personality and subjective well-being: focused on Big 5 personality factors and BAS/BIS. *Korean Journal of Psychological and Social Issues*.

[38] Cheung F. M., van de Vijver F. J. R., Leong F. T. L. (2011). Toward a new approach to the study of personality in culture. *American Psychologist*.

